# TRIM33 Reverses Cisplatin Resistance in Non-Small Cell Lung Cancer by Regulating the PI3K/AKT Pathway via Ubiquitination-Mediated Degradation of LPCAT1

**DOI:** 10.14740/wjon2729

**Published:** 2026-05-08

**Authors:** Jie Huang, Bao Qing Wang, Qin Wu, Li Ming Wang, Chao Guan

**Affiliations:** a Department of Respiratory Medicine, Shanghai Xuhui Central Hospital, Zhongshan-Xuhui Hospital, Fudan University, Xuhui, Shanghai 200031, China; b Department of Respiratory Medicine, Zhongshan Hospital, Fudan University, Xuhui, Shanghai 200031, China

**Keywords:** Non-small cell lung cancer, Cisplatin resistance, LPCAT1, TRIM33, Ubiquitination, PI3K/AKT signaling pathway, Glycolysis

## Abstract

**Background:**

Lung cancer is the leading cause of cancer-related deaths worldwide, with non-small cell lung cancer (NSCLC) accounting for 80–85% of cases. Cisplatin (DDP) is a first-line chemotherapy drug for NSCLC, but acquired DDP resistance severely limits therapeutic efficacy. Lysophosphatidylcholine acyltransferase 1 (LPCAT1) is involved in tumor progression, but its role in DDP resistance of NSCLC remains unclear. This study aimed to investigate the regulatory mechanism of LPCAT1 in DDP-resistant NSCLC and explore the potential role of tripartite motif-containing 33 (TRIM33) in modulating LPCAT1.

**Methods:**

DDP-resistant NSCLC cell lines (A549/DDP, PC-9/DDP) were established by gradual concentration gradient induction. Cell viability was detected by cell counting kit-8 (CCK-8) assay to determine half-maximal inhibitory concentration (IC_50_) and resistance index (RI). Glucose uptake, intracellular ATP, and lactate levels were measured to evaluate glycolytic activity. Quantitative real-time polymerase chain reaction (qRT-PCR) and Western blotting were used to detect mRNA and protein expression of LPCAT1, TRIM33, and PI3K/AKT pathway-related molecules. Flow cytometry was employed to analyze cell apoptosis. Immunoprecipitation and ubiquitination assays verified the interaction between TRIM33 and LPCAT1. *In vivo* xenograft models were established to confirm the regulatory role of LPCAT1 and TRIM33 in DDP resistance. Statistical analyses included Student’s *t*-test, one-way analysis of variance (ANOVA), and Student-Newman-Keuls test, with P < 0.05 considered statistically significant.

**Results:**

LPCAT1 protein expression was significantly upregulated in DDP-resistant NSCLC cells, while mRNA expression showed no significant difference. LPCAT1 overexpression enhanced DDP resistance, activated the PI3K/AKT signaling pathway, and promoted glycolysis in NSCLC cells, whereas LPCAT1 knockdown reversed these effects. TRIM33 expression was negatively correlated with DDP resistance, and TRIM33 directly interacted with LPCAT1 to promote its ubiquitination and degradation via the proteasomal pathway. Overexpression of TRIM33 inhibited the PI3K/AKT pathway and glycolysis, thereby sensitizing DDP-resistant cells to DDP. *In vivo* experiments confirmed that LPCAT1 promoted DDP resistance and tumor growth, while TRIM33 overexpression reversed this phenotype.

**Conclusion:**

TRIM33 regulates LPCAT1 stability through ubiquitination-mediated degradation, thereby suppressing PI3K/AKT signaling and glycolysis and attenuating DDP resistance in NSCLC. These findings suggest that the TRIM33-LPCAT1 axis may represent a potential therapeutic candidate for DDP-resistant NSCLC and provide a basis for further investigation of strategies to improve DDP sensitivity.

## Introduction

Lung cancer is the leading cause of cancer-related deaths globally, with increasing incidence and mortality in China, projected to rise by 40% from 2015 to 2030 [1, 2]. Non-small cell lung cancer (NSCLC) accounts for approximately 80–85% of all lung cancer cases, mainly including lung adenocarcinoma (LUAD) and lung squamous carcinoma (LUSC) [3, 4]. Despite advancements in diagnosis and treatment, the 5-year survival rate of NSCLC patients remains low [5]. Cisplatin (DDP) is the first-line chemotherapy drug for advanced NSCLC and NSCLC with wild-type epidermal growth factor receptor (EGFR) [6]. However, long-term chemotherapy inevitably induces DDP resistance, reducing treatment efficacy and patient survival. Elucidating the molecular mechanism of DDP resistance and identifying novel therapeutic targets are urgently needed.

Lysophosphatidylcholine acyltransferase 1 (LPCAT1) is a key enzyme in phosphatidylcholine (PC) biosynthesis, catalyzing the conversion of lysophosphatidylcholine (LPC) to PC. LPCAT1 is constitutively expressed in lung tissue, predominantly in type II alveolar cells, and is critical for lung function [7]. Accumulating evidence shows that LPCAT1 is overexpressed in various cancers, including hepatocellular carcinoma, colorectal cancer, and breast cancer, and promotes tumor growth, metastasis, and progression [8, 9]. In LUAD, LPCAT1 accelerates cell proliferation by activating the MYC-mediated PI3K/AKT signaling pathway [10] and contributes to gefitinib resistance via the EGFR/PI3K/AKT pathway [11]. The PI3K/AKT/mammalian target of rapamycin (mTOR) pathway regulates cell proliferation, survival, and metabolism, and its activation is closely associated with DDP resistance in lung cancer [12–18]. Inhibiting AKT1 or using PI3K inhibitors can enhance the sensitivity of DDP-resistant lung cancer cells to DDP [18], suggesting that the PI3K/AKT pathway may be a critical mediator of DDP resistance. However, the role of LPCAT1 in DDP-resistant NSCLC and its association with the PI3K/AKT pathway remain unclear.

Tripartite motif-containing 33 (TRIM33), also known as transcriptional intermediary factor 1γ (TIF-1γ), belongs to the TRIM family of E3 ubiquitin ligases, containing conserved RING, B-box, and coiled-coil domains, as well as a plant homeodomain (PHD) finger and bromodomain [19]. TRIM33 is frequently mutated, translocated, or downregulated in various cancers, including hepatocellular carcinoma, pancreatic cancer, and chronic myelomonocytic leukemia [20–22]. As a tumor suppressor, TRIM33 catalyzes the ubiquitination and degradation of nuclear β-catenin to inhibit WNT signaling in glioma and intestinal cancer [23, 24], and its loss contributes to bromodomain and extra-terminal domain inhibitor (BETi) resistance in colon cancer [25]. However, whether TRIM33 participates in DDP resistance of NSCLC by regulating target proteins has not been reported.

In this study, we established DDP-resistant NSCLC cell lines and explored the role of LPCAT1 in DDP resistance. We further investigated the regulatory relationship between TRIM33 and LPCAT1, and clarified the underlying molecular mechanisms involving the PI3K/AKT signaling pathway and glycolysis. Our findings provide new insights into overcoming DDP resistance in NSCLC and identify potential therapeutic targets.

## Materials and Methods

### Cell line and culture

Human NSCLC cell lines A549 and PC-9 were purchased from the American Type Culture Collection (ATCC, Manassas, USA). Cells were cultured in RPMI-1640 medium (Invitrogen, Life Technologies, USA) supplemented with 10% fetal bovine serum (FBS) and 1% penicillin/streptomycin, and maintained at 37 °C in a humidified incubator with 5% CO_2_.

### Establishment of DDP-resistant cell lines

DDP-resistant A549/DDP and PC-9/DDP cell lines were established by gradual induction with increasing DDP concentrations (Merck KGaA, Germany). Initially, cells were cultured in medium containing 0.1 mg/L DDP. The DDP concentration was gradually increased every 2 weeks until cells stably survived and proliferated in 5 mg/L DDP. After 4 months of induction and selection, the resistant cell lines were verified by cell counting kit-8 (CCK-8) assay.

### Drug sensitivity analysis

Cells were seeded in 96-well plates at a density of 5 × 10^3^ cells/well and incubated overnight. DDP was added at concentrations of 0, 0.5, 1, 5, 10, 20, 40, and 60 mg/L, with four replicates per concentration. After 72 h of incubation, 10 µL CCK-8 reagent (Dojindo Laboratories, Kumamoto, Japan) was added to each well, and absorbance at 450 nm was measured using a microplate reader. Cell viability (%) = (OD_450_ of experimental group/OD_450_ of control group) × 100. IC_50_ values were calculated using SPSS software, and the resistance index (RI) = IC_50_ of resistant cells/IC_50_ of parental cells.

### Cell proliferation assay

Cell proliferation was assessed using the CCK-8 (Beyotime Institute of Biotechnology, Shanghai, China). Exponentially growing cells were seeded in 96-well plates at a density of 2,000 cells per well and incubated overnight at 37 °C. Following the initial incubation, the cells were treated with 5 µM DDP. To evaluate the dynamic changes in cell proliferative capacity, the CCK-8 assay was performed at 0, 24, 48, and 72 h after DDP treatment. At each time point, CCK-8 reagent (10 µL) was added to each well and incubated for 4 h at 37 °C. The absorbance (OD value) was subsequently measured at 450 nm using a microplate reader (Thermo Fisher Scientific). Each group included six replicates, and the entire experiment was performed independently three times.

### Flow cytometry for apoptosis detection

Cells were digested with trypsin without ethylenediaminetetraacetic acid (EDTA), resuspended in phosphate-buffered saline (PBS), and centrifuged at 800 rpm at 4 °C for 10 min. The cell pellet was resuspended in binding buffer, and 5 µL annexin V-fluorescein isothiocyanate (FITC) and 5 µL propidium iodide (PI; Life Technologies, USA) were added. After incubation at room temperature for 15 min in the dark, apoptotic rates were detected using a flow cytometer (Beckman Coulter, USA).

### Glucose uptake assay

Glucose uptake was measured using the fluorescent glucose analog 2-[N-(7-nitrobenz-2-oxa-1,3-diazol-4-yl) amino]-2-deoxy-D-glucose (2-NBDG; Invitrogen, USA). Cells were cultured overnight, pretreated with glucose-free Krebs-Ringer bicarbonate (KRB) buffer (129 mM NaCl, 5 mM NaHCO_3_, 4.8 mM KCl, 1.2 mM KH_2_PO_4_, 1.0 mM CaCl_2_, 1.2 mM MgSO_4_, 10 mM HEPES, 0.1% BSA, pH 7.4) at 37 °C for 15 min, and then incubated in KRB buffer containing 400 µM 2-NBDG and 3.3 mM glucose at 37 °C for 10 min. After washing with KRB buffer, intracellular fluorescence intensity was detected using a fluorescence microplate reader.

### Biochemical testing

Intracellular ATP levels were determined using an ATP detection kit (Beyotime Biotechnology, Shanghai, China) according to the manufacturer’s instructions. Intracellular L-lactate levels were measured using an L-lactate assay kit (Beyotime Biotechnology, Shanghai, China) following the protocol. Absorbance was detected with a microplate reader, and concentrations were calculated based on standard curves.

### Quantitative real-time polymerase chain reaction (qRT-PCR)

Total RNA was extracted using TRIzol^®^ reagent (Invitrogen; Thermo Fisher Scientific, Inc., USA). Complementary DNA (cDNA) was synthesized using the SuperScript™ IV CellsDirect™ cDNA Synthesis Kit (Thermo Fisher Scientific, Inc., USA) with the following program: 42 °C for 60 min, 70 °C for 15 min, and cooling to 4 °C. qPCR was performed using SYBR^®^ Premix Ex Taq™ (DRR041A; Takara Bio, Inc., Japan) on an Applied Biosystems thermal cycler. The primer sequences are listed in Table 1. PCR parameters: initial denaturation at 94 °C for 3 min, followed by 40 cycles of 95 °C for 5 s, 65 °C for 35 s, and 72 °C for 60 s, with a final extension at 72 °C for 5 min. Relative gene expression was calculated using the 2^-ΔΔCT^ method, with GAPDH as the internal reference.

**Table 1:** Primer Sequences for qRT-PCR

Gene	Forward primer (5'-3')	Reverse primer (5'-3')
*LPCAT1*	5′-ATGGCAGCAGTCTTCTTCCTG-3′	5′-TCAGCAGCAGTCTTGTCCTT-3′
*TRIM33*	5′-AAGGTGAAGGTCGGAGTCAAC-3′	5′-TCAGCTGCTGCTGCTTACTT-3′
*GAPDH*	5′-GGCATGGACTGTGGTCATGAG-3′	5′-TGCACCACCAACTGCTTAGC-3′

### Western blotting and immunoprecipitation

Cells were lysed in radioimmunoprecipitation assay (RIPA) buffer (Beyotime Biotechnology, Shanghai, China) containing a protease inhibitor cocktail (Thermo Scientific, Waltham, MA, USA) and phenylmethylsulfonyl fluoride (PMSF). Protein concentration was determined using a bicinchoninic acid (BCA) protein assay kit. Fifty micrograms of total protein was separated by sodium dodecyl sulfate-polyacrylamide gel electrophoresis (SDS-PAGE) and transferred to polyvinylidene fluoride (PVDF) membranes (Sigma, St Louis, MO, USA). Membranes were blocked with 5% non-fat milk for 1 h at room temperature, incubated with primary antibodies overnight at 4 °C, and then with horseradish peroxidase (HRP)-conjugated secondary antibodies for 1 h at room temperature. Protein bands were visualized using an enhanced chemiluminescence (ECL) detection system, and densitometric analysis was performed using Quantity One Software (Bio-Rad, USA). GAPDH was used as the internal control. Primary antibodies: LPCAT1 (16112-1-AP), TRIM33 (55374-1-AP), GAPDH (60004-1-Ig) (ProteinTech, Wuhan, China); AKT (4691), phosphorylated AKT (p-AKT; 4060), glucose transporter 1 (GLUT1; 73015) (Cell Signaling Technology, MA, USA).

For immunoprecipitation, A549/DDP cells overexpressing TRIM33 were lysed in IP lysis buffer. Cell lysates were incubated with anti-LPCAT1 or anti-TRIM33 antibody overnight at 4 °C, followed by incubation with protein A/G-agarose beads for 2 h at 4 °C. Beads were collected, washed three times with lysis buffer, and associated proteins were eluted, denatured with 2× SDS loading buffer, and analyzed by Western blotting.

### Ubiquitination assay

A549/DDP cells were co-transfected with Flag-LPCAT1, HA-ubiquitin, and Myc-TRIM33 plasmids or empty vectors. After 48 h of transfection, cells were treated with 10 µM MG132 (a proteasome inhibitor) for 6 h. Cell lysates were immunoprecipitated with anti-Flag antibody, and ubiquitinated LPCAT1 was detected by Western blotting with anti-HA antibody.

### *In vivo* xenograft assay

Six-week-old male BALB/c nu/nu nude mice (Hangzhou Ziyuan Experimental Animal Technology Co., Ltd., Hangzhou, China) were used for xenograft experiments. Tumor cell suspensions (6 × 10^6^ A549, A549/DDP, PC-9, or PC-9/DDP cells in 100 µL serum-free medium) were injected subcutaneously into the right posterior flank of the mice. Tumor volume was measured once every 3 days using the formula: 0.5 × long diameter × short diameter^2^. When the tumor volume reached over 100 mm^3^, mice were intraperitoneally injected with 20 mg/kg DDP twice a week. The experimental endpoint was reached when the tumor diameter exceeded 2 cm or complications occurred. Mice were euthanized by carbon dioxide inhalation, and tumors were isolated, weighed, and fixed for hematoxylin and eosin (H&E) staining, terminal deoxynucleotidyl transferase-mediated dUTP nick-end labeling (TUNEL) staining, Ki67 immunofluorescence staining, and Western blotting. All animal experiments were approved by the Institutional Animal Care and Use Committee of the Fudan University Experimental Animal Center (Approval No.: 2023-A012). All experimental protocols involving animals have been reviewed and approved by this board to ensure compliance with ethical standards. The study strictly adheres to all applicable institutional ethical guidelines and complies with the requirements of the Guide for the Care and Use of Laboratory Animals, aiming to minimize animal suffering and safeguard the welfare and rights of experimental animals.

### Statistical analysis

All data are presented as mean ± standard deviation (SD). Before applying parametric tests, normality was assessed using the Shapiro–Wilk test, and homogeneity of variance was evaluated using Levene’s test. Comparisons between two groups were performed using Student’s *t*-test, while differences among multiple groups were analyzed using one-way analysis of variance (ANOVA) followed by the Student–Newman–Keuls (SNK) *post hoc* test. Each experiment was independently repeated at least three times. Statistical analyses were performed using SPSS 22.0 software, and P < 0.05 was considered statistically significant.

## Results

### Upregulation of LPCAT1 protein expression in DDP-resistant NSCLC cells

**Figure 1:**
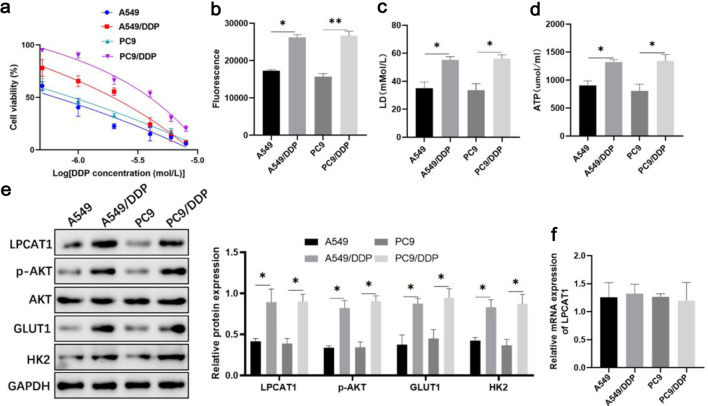
Changes in LPCAT1, p-AKT, and glycolysis-related molecule expression in DDP-resistant lung cancer cells. (a) Viability of A549, A549/DDP, PC-9, and PC-9/DDP cells treated with DDP. (b) Intracellular glucose uptake. (c) Intracellular lactate levels. (d) Intracellular ATP levels. (e) Western blot analysis of LPCAT1, p-AKT, GLUT1, and HK2 protein expression. (f) qRT-PCR analysis of LPCAT1 mRNA expression. Data are presented as the mean ± SD (n = 3). *P < 0.05, **P < 0.01 vs. parental cells.

DDP-resistant cell lines (A549/DDP, PC-9/DDP) were successfully established, as confirmed by CCK-8 assay. The IC_50_ values of A549/DDP (21.15 µM) and PC-9/DDP (32.26 µM) were more than five times higher than those of parental A549 (3.74 µM) and PC-9 (5.37 µM) cells (Fig. 1a). To confirm the resistance phenotype of the established cell lines, we performed a time-course cell proliferation assay under 5 µM DDP treatment. The parental A549 and PC-9 cells exhibited marked growth inhibition and a significant decrease in cell viability starting from 48 h, the DDP-resistant sublines (A549/DDP and PC-9/DDP) remained highly proliferative. Specifically, at 72 h, the OD450 values of resistant cells were significantly higher than those of the sensitive counterparts, demonstrating the stable maintenance of chemoresistance in the resistant sublines (Supplementary Material 1, wjon.elmerpub.com). Glycolytic activity was significantly enhanced in DDP-resistant cells, as evidenced by increased glucose uptake, lactate production, and ATP levels (Fig. 1b–d). Western blotting showed that LPCAT1, p-AKT, GLUT1, and hexokinase 2 (HK2) protein levels were upregulated in A549/DDP and PC-9/DDP cells, while qRT-PCR revealed no significant difference in LPCAT1 mRNA expression between resistant and parental cells (Fig. 1e, f), indicating post-transcriptional regulation of LPCAT1.

### LPCAT1 promotes DDP resistance by activating the PI3K/AKT pathway and glycolysis

**Figure 2:**
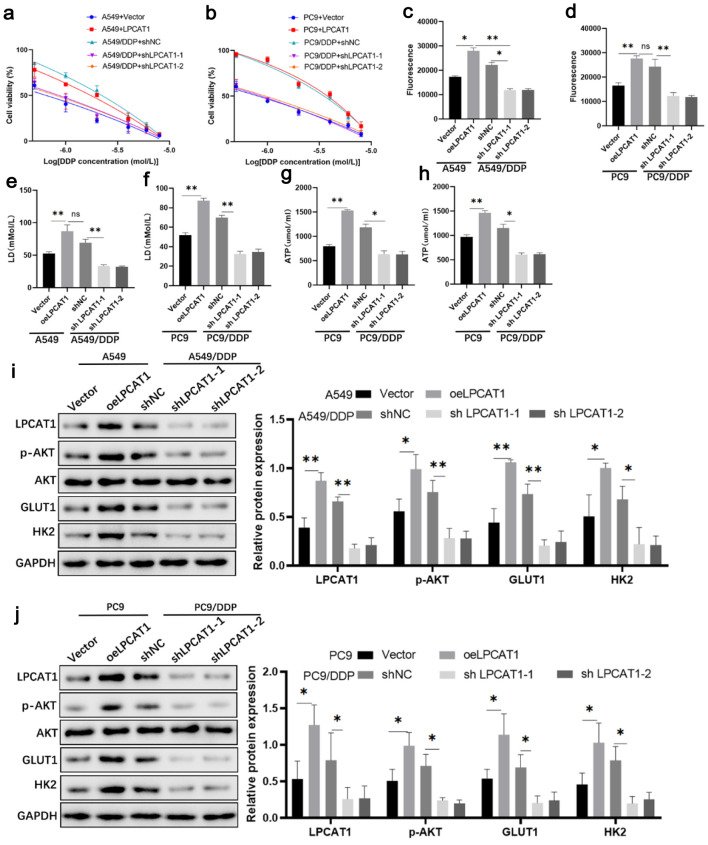
Effect of LPCAT1 expression on cell drug resistance. (a) Viability of A549 and A549/DDP cells treated with DDP. (b) Viability of PC-9 and PC-9/DDP cells treated with DDP. (c) Glucose uptake in A549 and A549/DDP cells. (d) Glucose uptake in PC-9 and PC-9/DDP cells. (e) Lactate levels in A549 and A549/DDP cells. (f) Lactate levels in PC-9 and PC-9/DDP cells. (g) ATP levels in A549 and A549/DDP cells. (h) ATP levels in PC-9 and PC-9/DDP cells. (i) Western blot analysis of LPCAT1, p-AKT, GLUT1, and HK2 expression in A549 and A549/DDP cells. (j) Western blot analysis of LPCAT1, p-AKT, GLUT1, and HK2 expression in PC-9 and PC-9/DDP cells. Data are presented as the mean ± SD (n = 3). *P < 0.05, **P < 0.01 vs. control group.

CCK-8 assay showed that LPCAT1 overexpression reduced DDP sensitivity in A549 and PC-9 cells, while LPCAT1 knockdown by short hairpin RNA (shRNA) enhanced DDP sensitivity in A549/DDP and PC-9/DDP cells (Fig. 2a, b). LPCAT1 overexpression promoted DDP resistance in parental A549 and PC-9 cells, while its knockdown abrogated the resistant trait in DDP-resistant sublines (Supplementary Material 2, wjon.elmerpub.com). Flow cytometry showed that LPCAT1 knockdown increased the apoptotic rate of DDP-treated resistant cells (Supplementary Material 3, wjon.elmerpub.com). LPCAT1 overexpression significantly increased glucose uptake, lactate production, ATP levels, and the expression of p-AKT, GLUT1, and HK2 in parental cells, whereas LPCAT1 knockdown reversed these effects in resistant cells (Fig. 2c–j). These results indicate that LPCAT1 promotes DDP resistance by activating the PI3K/AKT pathway and glycolysis.

**Figure 3:**
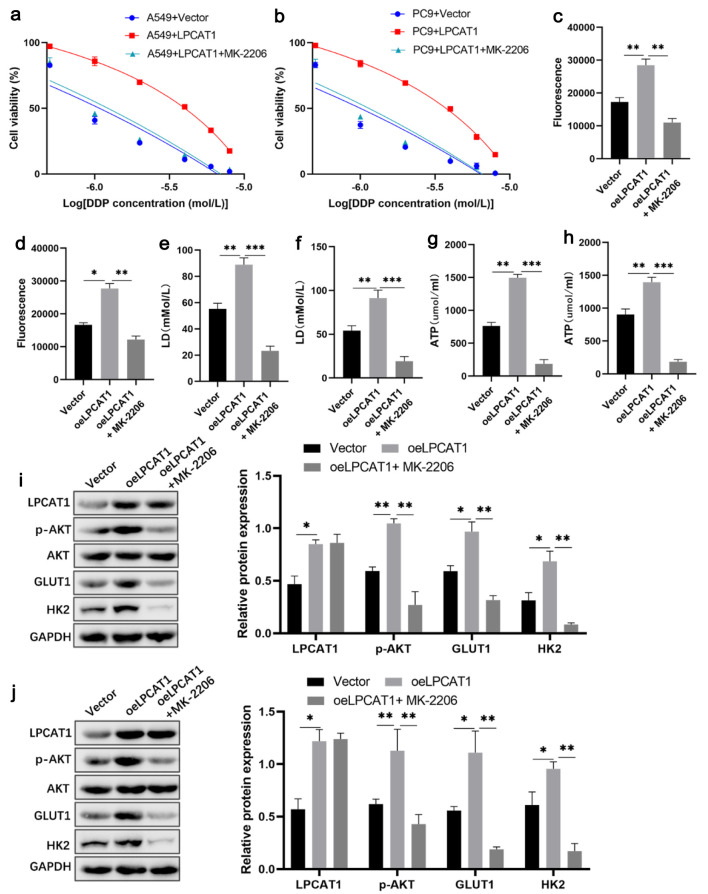
Inhibition of AKT signaling reverses LPCAT1-mediated cisplatin resistance and glycolysis in NSCLC cells. (a) Viability of A549 cells treated with DDP. (b) Viability of PC-9 cells treated with DDP. (c) Glucose uptake in A549 cells. (d) Glucose uptake in PC-9 cells. (e) Lactate levels in A549 cells. (f) Lactate levels in PC-9 cells. (g) ATP levels in A549 cells. (h) ATP levels in PC-9 cells. (i) Western blot analysis of LPCAT1, p-AKT, GLUT1, and HK2 expression in A549 cells. (j) Western blot analysis of LPCAT1, p-AKT, GLUT1, and HK2 expression in PC-9 cells. Data are presented as the mean ± SD (n = 3). *P < 0.05, **P < 0.01 vs. control group.

To further elucidate whether LPCAT1-mediated DDP resistance is dependent on the PI3K/AKT signaling pathway, LPCAT1-overexpressing A549 and PC-9 cells were treated with the highly specific AKT inhibitor MK-2206. As expected, MK-2206 treatment effectively reversed the LPCAT1-induced enhancement of DDP resistance and glycolysis (Fig. 3a–h). Furthermore, the upregulation of p-AKT, GLUT1, and HK2 driven by LPCAT1 overexpression was markedly abrogated by MK-2206 (Fig. 3i, j). Flow cytometry analysis additionally revealed that MK-2206 restored the sensitivity to DDP-induced apoptosis in LPCAT1-overexpressing A549 and PC-9 cells (Supplementary Material 4, wjon.elmerpub.com).

### *In vivo* validation of LPCAT1-mediated DDP resistance

**Figure 4:**
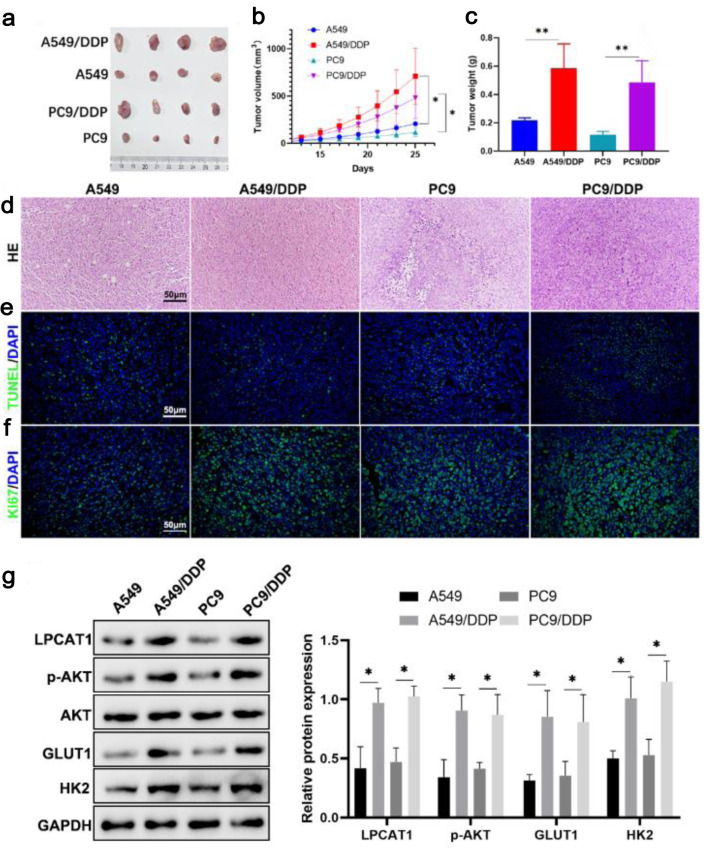
*In vivo* experimental analysis showing that LPCAT1 promotes lung cancer drug resistance by regulating AKT and glycolysis. (a) Photos of xenograft tumors. (b) Tumor growth curves. (c) Weight of xenograft tumors. (d) H&E staining showing pathological changes in tumors (scale bar: 100 µm). (e) TUNEL staining showing tumor cell apoptosis (scale bar: 100 µm). (f) Ki67 staining showing tumor cell proliferation (scale bar: 100 µm). (g) Western blot analysis of LPCAT1, p-AKT, GLUT1, and HK2 expression in PC-9 and PC-9/DDP tumor tissues. Data are presented as the mean ± SD (n = 6). *P < 0.05, **P < 0.01 vs. parental cell groups.

In xenograft models, mice inoculated with A549/DDP or PC-9/DDP cells developed significantly larger tumors than those inoculated with parental cells after DDP treatment (Fig. 4a–c). H&E staining showed pathological damage (necrosis, vacuolization) in tumors from parental cell groups, while tumors from resistant cell groups had dense and ordered cell arrangements (Fig. 4d). TUNEL staining revealed a lower apoptotic rate, and Ki67 staining showed a higher proliferation rate in resistant cell-derived tumors (Fig. 4e, f). Western blotting confirmed that LPCAT1, p-AKT, GLUT1, and HK2 protein levels were higher in tumors from resistant cell groups (Fig. 4g). These results confirm that LPCAT1 promotes DDP resistance and tumor growth *in vivo* by activating the PI3K/AKT pathway and glycolysis.

### TRIM33 expression is negatively correlated with DDP resistance

**Figure 5:**
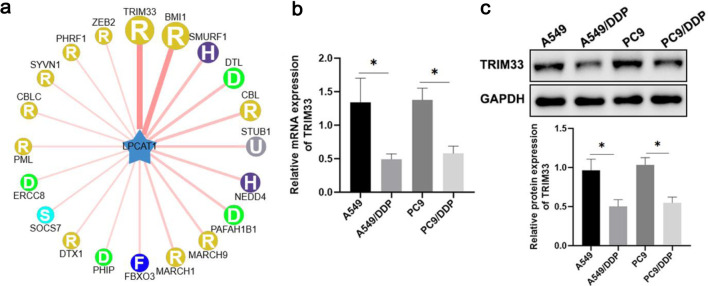
TRIM33 affects lung cancer drug resistance by regulating LPCAT1 expression. (a) UbiBrowser database analysis showing that TRIM33 ubiquitinates LPCAT1. (b) TRIM33 mRNA expression levels in A549, PC-9, A549/DDP, and PC-9/DDP cells. (c) TRIM33 protein expression levels in A549, PC-9, A549/DDP, and PC-9/DDP cells. Data are presented as the mean ± SD (n = 3). *P < 0.05, **P < 0.01 vs. parental cells.

UbiBrowser database analysis predicted that TRIM33 could ubiquitinate LPCAT1 (Fig. 5a). qRT-PCR and Western blotting showed that TRIM33 mRNA and protein expression were significantly lower in DDP-resistant cells than in parental cells, indicating a negative correlation between TRIM33 expression and DDP resistance (Fig. 5b, c).

### TRIM33 sensitizes DDP-resistant cells to DDP by inhibiting the PI3K/AKT pathway and glycolysis

**Figure 6:**
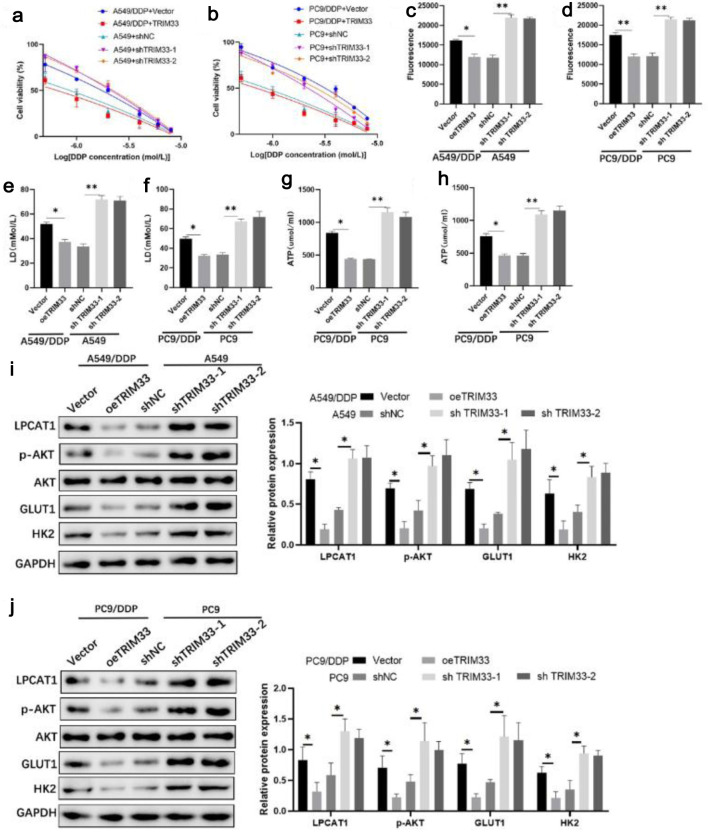
Effect of TRIM33 expression on cell drug resistance. (a) Viability of A549 and A549/DDP cells treated with DDP. (b) Viability of PC-9 and PC-9/DDP cells treated with DDP. (c) Glucose uptake in A549 and A549/DDP cells. (d) Glucose uptake in PC-9 and PC-9/DDP cells. (e) Lactate levels in A549 and A549/DDP cells. (f) Lactate levels in PC-9 and PC-9/DDP cells. (g) ATP levels in A549 and A549/DDP cells. (h) ATP levels in PC-9 and PC-9/DDP cells. (i) Western blot analysis of LPCAT1, p-AKT, GLUT1, and HK2 expression in A549 and A549/DDP cells. (j) Western blot analysis of LPCAT1, p-AKT, GLUT1, and HK2 expression in PC-9 and PC-9/DDP cells. Data are presented as the mean ± SD (n = 3). *P < 0.05, **P < 0.01 vs. control group.

CCK-8 assay showed that TRIM33 knockdown reduced DDP sensitivity in parental cells, while TRIM33 overexpression enhanced DDP sensitivity in resistant cells (Fig. 6a, b). Overexpression of TRIM33 significantly re-sensitized DDP-resistant cells to DDP, whereas TRIM33 knockdown effectively conferred chemoresistance to the sensitive parental NSCLC cells (Supplementary Material 5, wjon.elmerpub.com). Flow cytometry showed that overexpression of TRIM33 in A549/DDP and PC-9/DDP cells dramatically increased the rate of DDP-induced apoptosis. In contrast, knockdown of TRIM33 in the parental A549 and PC-9 cells significantly protected them from DDP-induced cell death (Supplementary Material 6, wjon.elmerpub.com). TRIM33 overexpression decreased glucose uptake, lactate production, ATP levels, and the expression of p-AKT, GLUT1, and HK2 in resistant cells, whereas TRIM33 knockdown increased these parameters in parental cells (Fig. 6c–j). These results suggest that TRIM33 modulates DDP resistance by regulating the PI3K/AKT pathway and glycolysis.

**Figure 7:**
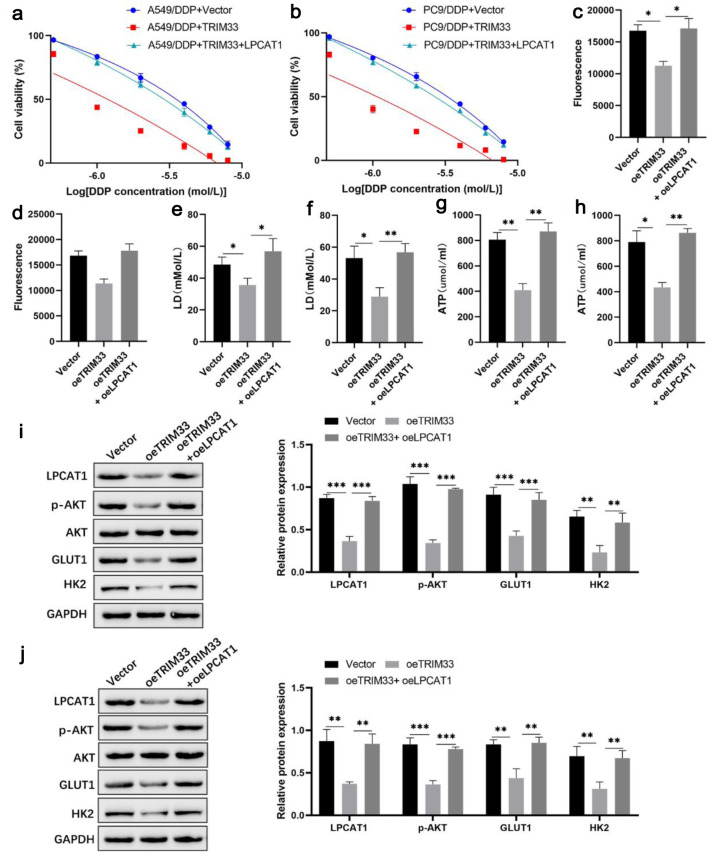
LPCAT1 overexpression rescues TRIM33-induced cisplatin sensitization in NSCLC cells. (a) Viability of A549/DDP cells treated with DDP. (b) Viability of PC-9/DDP cells treated with DDP. (c) Glucose uptake in A549/DDP cells. (d) Glucose uptake in PC-9/DDP cells. (e) Lactate levels in A549/DDP cells. (f) Lactate levels in PC-9/DDP cells. (g) ATP levels in A549/DDP cells. (h) ATP levels in PC-9/DDP cells. (i) Western blot analysis of LPCAT1, p-AKT, GLUT1, and HK2 expression in A549/DDP cells. (j) Western blot analysis of LPCAT1, p-AKT, GLUT1, and HK2 expression in PC-9/DDP cells. Data are presented as the mean ± SD (n = 3). *P < 0.05, **P < 0.01 vs. control group.

To determine whether TRIM33 sensitizes NSCLC cells to DDP in an LPCAT1-dependent manner, we performed rescue experiments in DDP-resistant cells. As previously observed, ectopic expression of TRIM33 in A549/DDP and PC-9/DDP cells severely impaired cell proliferation and exacerbated DDP-induced apoptosis. Strikingly, the simultaneous re-introduction of LPCAT1 effectively abrogated the tumor-suppressive effects of TRIM33. Specifically, LPCAT1 overexpression restored the proliferative capacity of these cells and significantly reduced the elevated apoptosis rate caused by TRIM33 under DDP stress (Fig. 7 and Supplementary Material 7, wjon.elmerpub.com).

### TRIM33 promotes LPCAT1 degradation through ubiquitination

**Figure 8:**
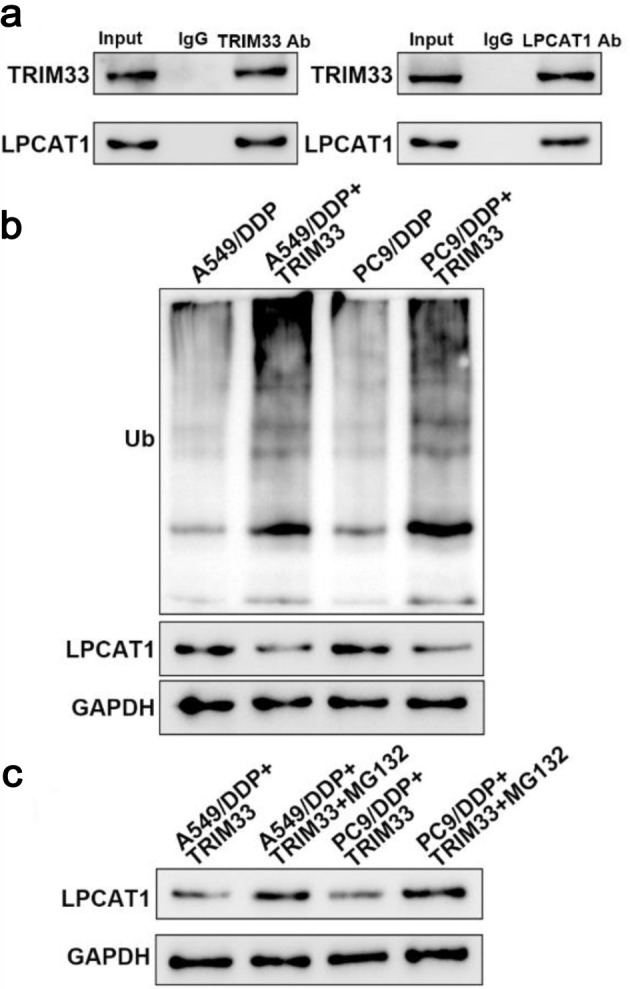
TRIM33 promotes LPCAT1 degradation through ubiquitination. (a) Immunoprecipitation analysis of the interaction between TRIM33 and LPCAT1. (b) Detection of LPCAT1 ubiquitination levels. (c) Effect of MG132 on LPCAT1 ubiquitination levels.

Immunoprecipitation assay confirmed a direct interaction between TRIM33 and LPCAT1 in A549/DDP cells overexpressing TRIM33 (Fig. 8a). Ubiquitination assay showed that TRIM33 overexpression increased LPCAT1 ubiquitination (Fig. 8b). Treatment with MG132 inhibited TRIM33-mediated LPCAT1 degradation (Fig. 8c). These results indicate that TRIM33 promotes LPCAT1 ubiquitination and degradation via the proteasomal pathway.

## Discussion

DDP is widely used in the treatment of various tumors, including lung cancer, but acquired resistance remains a major clinical challenge [26, 27]. Most malignant tumors, except testicular cancer, gradually develop DDP resistance, reducing therapeutic efficacy [28]. Elucidating the mechanism of DDP resistance is crucial for improving the prognosis of NSCLC patients. In this study, we found that LPCAT1 protein expression is significantly upregulated in DDP-resistant NSCLC cells, and LPCAT1 promotes DDP resistance by activating the PI3K/AKT signaling pathway and glycolysis. TRIM33 acts as an E3 ubiquitin ligase to promote LPCAT1 ubiquitination and degradation, thereby inhibiting the PI3K/AKT pathway and glycolysis and reversing DDP resistance.

LPCAT1 is a key enzyme in PC biosynthesis and is overexpressed in various cancers, promoting tumor progression, metastasis, and recurrence [8, 9, 29, 30]. However, its role in DDP-resistant NSCLC has not been reported. Our study is the first to show that LPCAT1 protein expression is upregulated in DDP-resistant NSCLC cells, while mRNA expression is unchanged, indicating post-transcriptional regulation. LPCAT1 overexpression enhances DDP resistance in parental NSCLC cells, while LPCAT1 knockdown sensitizes resistant cells to DDP and inhibits tumor growth in xenograft models. Together, these findings support a role for LPCAT1 in the development and maintenance of DDP resistance in NSCLC.

The PI3K/AKT pathway is frequently activated in cancers and regulates cell proliferation, survival, invasion, and metabolism [31, 32]. Activation of the PI3K/AKT pathway is closely associated with DDP resistance in NSCLC [13, 18, 33]. For example, AFAP1-AS1 interacts with EZH2 to activate the PI3K/AKT pathway, leading to DDP resistance [33]. Our study shows that LPCAT1 overexpression increases p-AKT expression, while LPCAT1 knockdown inhibits p-AKT expression in DDP-resistant cells, suggesting that LPCAT1 promotes DDP resistance by activating the PI3K/AKT pathway. Additionally, the PI3K/AKT pathway can enhance glucose transporter activity and promote glycolysis [17], which is consistent with our findings that LPCAT1 regulates glycolytic activity in NSCLC cells. Glycolysis provides energy and metabolic intermediates for tumor cell proliferation and survival, and enhanced glycolysis is a common feature of drug-resistant tumor cells [17]. Our results show that LPCAT1 overexpression increases glucose uptake, lactate production, and ATP levels, while LPCAT1 knockdown reverses these effects, indicating that LPCAT1 promotes DDP resistance by enhancing glycolysis.

TRIM33 belongs to the TIF1 family of E3 ubiquitin ligases and functions as a tumor suppressor in various cancers [19, 34, 35]. For example, TRIM33 inhibits lung adenocarcinoma metastasis by interacting with the TAF15/TBP complex [34], and reduced TRIM33 expression promotes hepatocellular carcinoma metastasis [35]. In clear cell renal cell carcinoma (ccRCC), miR-629 targets TRIM33 to promote TGFβ/Smad signaling and metastasis [36]. However, the role of TRIM33 in DDP-resistant NSCLC remains unclear. Our study predicts that TRIM33 can ubiquitinate LPCAT1 via the UbiBrowser database, and we confirm that TRIM33 directly interacts with LPCAT1 to promote its ubiquitination and degradation via the proteasomal pathway. TRIM33 expression is downregulated in DDP-resistant cells, and TRIM33 overexpression sensitizes resistant cells to DDP by inhibiting the PI3K/AKT pathway and glycolysis. These results indicate that TRIM33 modulates DDP resistance by regulating LPCAT1 stability.

Although our study provides mechanistic evidence supporting the involvement of the TRIM33-LPCAT1-PI3K/AKT axis in DDP resistance, several limitations should be acknowledged. First, the specific ubiquitination site(s) of LPCAT1 targeted by TRIM33 were not identified, and further mutational analyses are required to define the relevant lysine residues. Second, the clinical relevance of LPCAT1 and TRIM33 expression remains to be validated in patient-derived samples and well-annotated clinical cohorts, particularly those with matched DDP response data. Third, the potential therapeutic value of targeting this axis should be interpreted with caution, as our current evidence is primarily based on *in vitro* models and xenograft experiments. Additional studies using patient-derived models and clinical specimens will be necessary to determine whether LPCAT1 may serve as a robust biomarker or therapeutic candidate in DDP-resistant NSCLC. Finally, the potential synergistic effect of TRIM33 overexpression and DDP treatment *in vivo* warrants further investigation.

### Conclusions

In conclusion, our study demonstrates that LPCAT1 is upregulated in DDP-resistant NSCLC cells and promotes DDP resistance by activating the PI3K/AKT signaling pathway and glycolysis. TRIM33 acts as an E3 ubiquitin ligase to promote LPCAT1 ubiquitination and degradation, thereby reversing DDP resistance in NSCLC. These findings identify the TRIM33-LPCAT1-PI3K/AKT axis as a potentially important regulatory mechanism in DDP-resistant NSCLC. While this pathway may represent a promising direction for future investigation, further validation in patient-derived models and clinical samples is required before its translational or therapeutic significance can be established.

## Supplementary Material

Suppl 1 Proliferation of parental and DDP-resistant NSCLC cells under 5 µM cisplatin treatment.

Suppl 2 LPCAT1 modulates cisplatin resistance in A549, A549/DDP, PC-9, and PC-9/DDP cells.

Suppl 3 LPCAT1 inhibits cisplatin-induced apoptosis in NSCLC cells.

Suppl 4 MK-2206 reverses LPCAT1-mediated inhibition of cisplatin-induced apoptosis.

Suppl 5 TRIM33 modulates cisplatin resistance in A549, A549/DDP, PC-9, and PC-9/DDP cells.

Suppl 6 RIM33 enhances cisplatin-induced apoptosis in NSCLC cells.

Suppl 7 LPCAT1 overexpression restores cisplatin resistance and offsets TRIM33-induced apoptosis.

## Data Availability

The datasets used and/or analyzed during the current study are available from the corresponding author upon reasonable request.

## Abbreviations

ANOVA: analysis of variance
ATCC: American Type Culture Collection
BETi: bromodomain and extra-terminal domain inhibitor
BSA: bovine serum albumin
CCK-8: cell counting kit-8
ccRCC: clear cell renal cell carcinoma
cDNA: complementary DNA
DDP: cisplatin
EDTA: ethylenediaminetetraacetic acid
EGFR: epidermal growth factor receptor
FBS: fetal bovine serum
FITC: fluorescein isothiocyanate
GAPDH: glyceraldehyde-3-phosphate dehydrogenase
GLUT1: glucose transporter 1
H&E: hematoxylin and eosin
HEPES: 4-(2-hydroxyethyl)piperazine-1-ethanesulfonic acid
HK2: hexokinase 2
HRP: horseradish peroxidase
IC_50_: half-maximal inhibitory concentration
IP: immunoprecipitation
KRB: Krebs-Ringer bicarbonate
LPC: lysophosphatidylcholine
LPCAT1: lysophosphatidylcholine acyltransferase 1
LUAD: lung adenocarcinoma
LUSC: lung squamous carcinoma
MAST1: microtubule-associated serine/threonine kinase 1
MG132: carbobenzoxy-Leu-Leu-leucinal
mTOR: mammalian target of rapamycin
2-NBDG: 2-[N-(7-nitrobenz-2-oxa-1,3-diazol-4-yl) amino]-2-deoxy-D-glucose
NSCLC: non-small cell lung cancer
p-AKT: phosphorylated AKT
PBS: phosphate-buffered saline
PC: phosphatidylcholine
PI: propidium iodide
PI3K/AKT: phosphatidylinositol 3-kinase/protein kinase B
PVDF: polyvinylidene fluoride
qRT-PCR: quantitative real-time polymerase chain reaction
RI: resistance index
SD: standard deviation
SDS-PAGE: sodium dodecyl sulfate-polyacrylamide gel electrophoresis
shRNA: short hairpin RNA
TGFβ/Smad: transforming growth factor β/Smad
TIF-1γ: transcriptional intermediary factor 1γ
TRIM33: tripartite motif-containing 33
TUNEL: terminal deoxynucleotidyl transferase-mediated dUTP nick-end labeling
USP1: ubiquitin-specific protease 1
