# Supplementary material for: TRIM33 Reverses Cisplatin Resistance in Non-Small Cell Lung Cancer by Regulating the PI3K/AKT Pathway via Ubiquitination-Mediated Degradation of LPCAT1
Source: World J Oncol. 2026 May 8;17(3):366–79. doi: 10.14740/wjon2729 (PMC13171270; doi:10.14740/wjon2729)
Supplement: Suppl 1 — Proliferation of parental and DDP-resistant NSCLC cells under 5 µM cisplatin treatment. [file wjon-17-03-366-s001.docx]

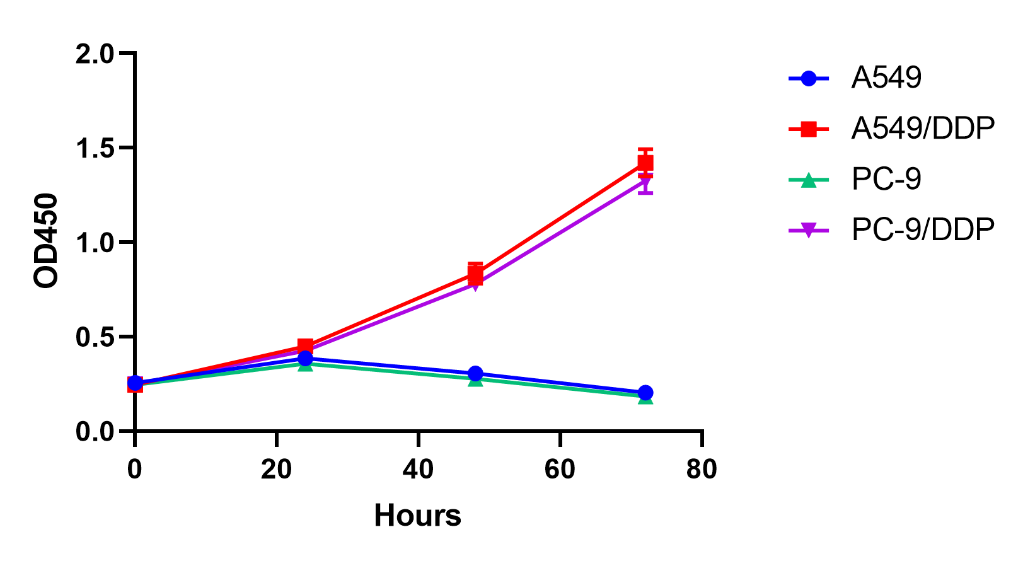


**Suppl 1.** Proliferation of parental and DDP-resistant NSCLC cells under 5μM cisplatin treatment. Data are presented as Mean ± SD (n=6).
