# Supplementary material for: TRIM33 Reverses Cisplatin Resistance in Non-Small Cell Lung Cancer by Regulating the PI3K/AKT Pathway via Ubiquitination-Mediated Degradation of LPCAT1
Source: World J Oncol. 2026 May 8;17(3):366–79. doi: 10.14740/wjon2729 (PMC13171270; doi:10.14740/wjon2729)
Supplement: Suppl 2 — LPCAT1 modulates cisplatin resistance in A549, A549/DDP, PC-9, and PC-9/DDP cells. [file wjon-17-03-366-s002.docx]

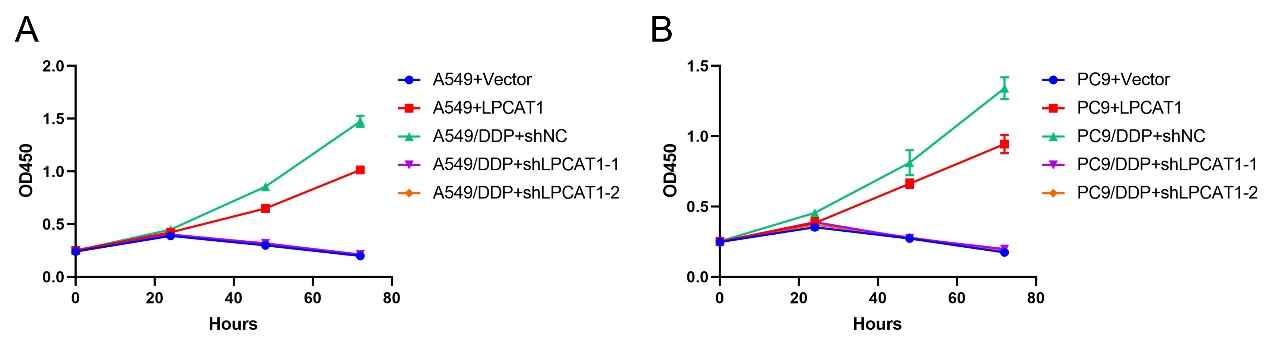


**Suppl 2.** LPCAT1 modulates cisplatin resistance in A549, A549/DDP, PC-9, and PC-9/DDP cells.

(A) Growth curves of parental A549 cells following LPCAT1 overexpression, and A549/DDP cells after LPCAT1 knockdown. (B) Growth curves of parental PC-9 cells following LPCAT1 overexpression, and PC-9/DDP cells after LPCAT1 knockdown. Data are presented as Mean ± SD (n=6).
