# Supplementary material for: TRIM33 Reverses Cisplatin Resistance in Non-Small Cell Lung Cancer by Regulating the PI3K/AKT Pathway via Ubiquitination-Mediated Degradation of LPCAT1
Source: World J Oncol. 2026 May 8;17(3):366–79. doi: 10.14740/wjon2729 (PMC13171270; doi:10.14740/wjon2729)
Supplement: Suppl 6 — RIM33 enhances cisplatin-induced apoptosis in NSCLC cells. [file wjon-17-03-366-s006.docx]

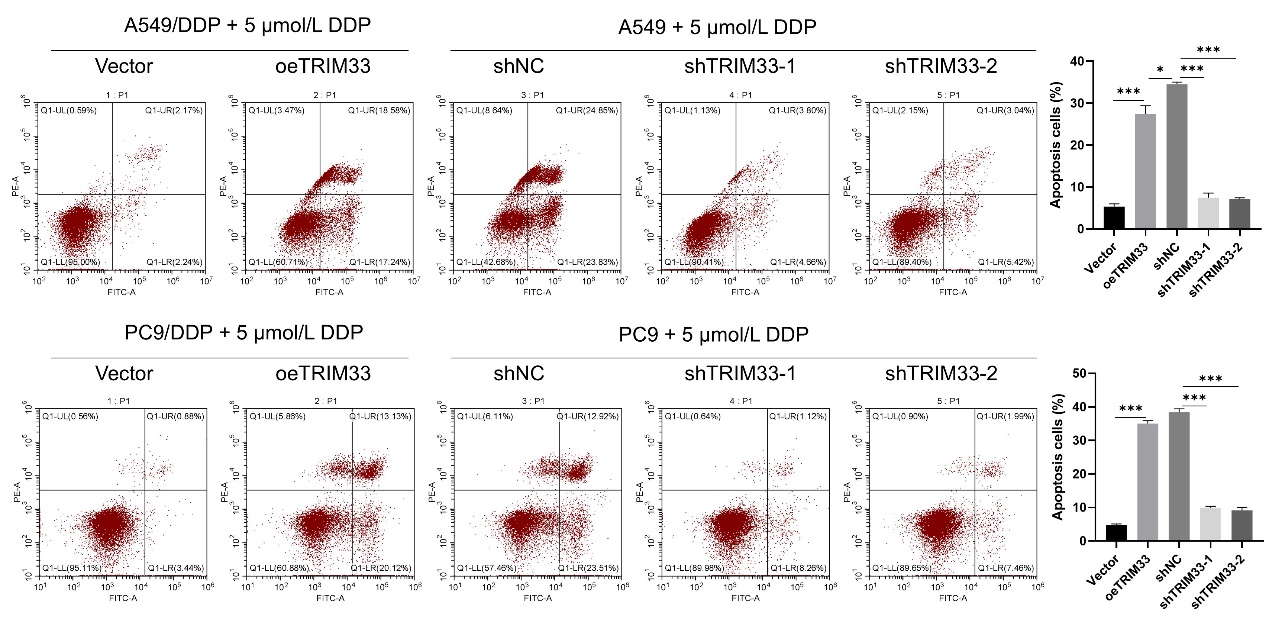


**Suppl 6.** TRIM33 enhances cisplatin-induced apoptosis in NSCLC cells. Data are presented as Mean ± SD (n=3). **P*<0.05; ****P*<0.001.
