# Supplementary material for: TRIM33 Reverses Cisplatin Resistance in Non-Small Cell Lung Cancer by Regulating the PI3K/AKT Pathway via Ubiquitination-Mediated Degradation of LPCAT1
Source: World J Oncol. 2026 May 8;17(3):366–79. doi: 10.14740/wjon2729 (PMC13171270; doi:10.14740/wjon2729)
Supplement: Suppl 7 — LPCAT1 overexpression restores cisplatin resistance and offsets TRIM33-induced apoptosis. [file wjon-17-03-366-s007.docx]

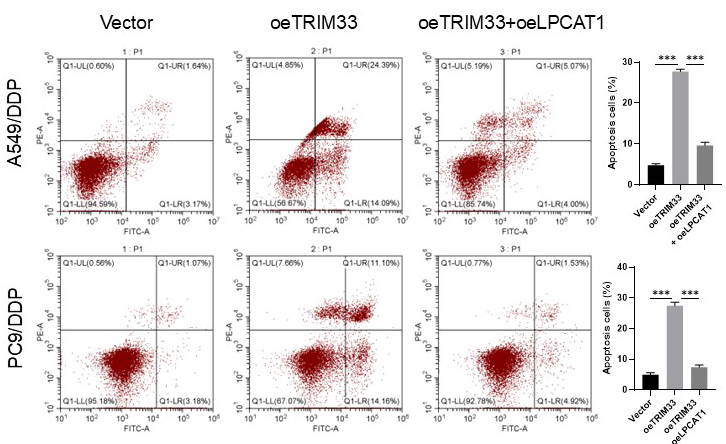


**Suppl 7.** LPCAT1 overexpression restores cisplatin resistance and offsets TRIM33-induced apoptosis. Data are presented as Mean ± SD (n=3). ***P<0.001.
